# Correction: “Timed Up & Go”: A Screening Tool for Predicting 30-Day Morbidity in Onco-Geriatric Surgical Patients? *A Multicenter Cohort Study*

**DOI:** 10.1371/journal.pone.0103907

**Published:** 2014-07-23

**Authors:** The PLOS ONE Staff

There is an error in the seventh author’s name. Nicola de’Liguori Carino’s last name is de’Liguori Carino, not Carino, as previously noted.
