# Correction: "Timed Up & Go": A Screening Tool for Predicting 30-Day Morbidity in Onco-Geriatric Surgical Patients? *A Multicenter Cohort Study*

**DOI:** 10.1371/journal.pone.0147993

**Published:** 2016-01-26

**Authors:** Monique G. Huisman, Barbara L. van Leeuwen, Giampaolo Ugolini, Isacco Montroni, John Spiliotis, Cesare Stabilini, Nicola de’Liguori Carino, Eriberto Farinella, Geertruida H. de Bock, Riccardo A. Audisio

## Notice of Republication

This article was republished on January 8, 2016, to correct errors in the values throughout the article as well as well as an error in the affiliations. Please download this article again to view the correct version. The originally published, uncorrected article and the republished, corrected article are provided here for reference.

## Supporting Information

S1 File Originally published, uncorrected article. (PDF)

S2 File Republished, corrected article. (PDF)
