# Supplementary material for: Correction: "Timed Up & Go": A Screening Tool for Predicting 30-Day Morbidity in Onco-Geriatric Surgical Patients? A Multicenter Cohort Study
Source: PLoS One. 2016 Jan 26;11(1):e0147993. doi: 10.1371/journal.pone.0147993 (PMC4727939; doi:10.1371/journal.pone.0147993)
Supplement: S2 File — (PDF) [file pone.0147993.s002.pdf]

PLOS | ONE

RESEARCH ARTICLE

"Timed Up & Go": A Screening Tool for Predicting 30-Day Morbidity in Onco-Geriatric Surgical Patients? *A Multicenter Cohort Study*

Monique G. Huisman[1]*, Barbara L. van Leeuwen[1], Giampaolo Ugolini[2], Isacco Montroni[2], John Spiliotis[3], Cesare Stabilini[4], Nicola de'Liguori Carino[5], Eriberto Farinella[6], Geertruida H. de Bock[7], Riccardo A. Audisio[8]

1 Department of Surgery, University of Groningen, University Medical Center Groningen, Groningen, The Netherlands, **2** Department of Surgery, S. Orsola Malpighi Hospital, Bologna, Italy, **3** Department of Surgery, Metaxa Cancer Hospital, Piraeus, Greece, and Regional University Hospital of Patras, Patras, Greece, **4** Department of Surgery, San Martino University Hospital, Genua, Italy, **5** Department of Surgery, North Manchester General Hospital, Manchester, United Kingdom, **6** Department of Surgery, S. Maria Hospital, Perugia, Italy, and Luton & Dunstable Hospital, Luton, United Kingdom, **7** Department of Epidemiology, University of Groningen, University Medical Center Groningen, Groningen, The Netherlands, **8** Department of Surgery, University of Liverpool St. Helens Teaching Hospital, St. Helens, United Kingdom

* m.g.huisman@umcg.nl

CrossMark
click for updates

OPEN ACCESS

Citation: Huisman MG, van Leeuwen BL, Ugolini G, Montroni I, Spiliotis J, Stabilini C, et al. (2014) "Timed Up & Go": A Screening Tool for Predicting 30-Day Morbidity in Onco-Geriatric Surgical Patients? *A Multicenter Cohort Study.* PLoS ONE 9(1): e0086863. doi:10.1371/journal.pone.0086863

Editor: Kazuaki Takabe, Virginia Commonwealth University School of Medicine, UNITED STATES

Received: April 10, 2013

Accepted: December 18, 2013

Published: January 24, 2014

Copyright: © 2014 Huisman et al. This is an open access article distributed under the terms of the Creative Commons Attribution License, which permits unrestricted use, distribution, and reproduction in any medium, provided the original author and source are credited.

Funding: The authors have no support or funding to report.

Competing Interests: The authors have declared that no competing interests exist.

Abstract

Objective

To determine the predictive value of the "Timed Up & Go" (TUG), a validated assessment tool, on a prospective cohort study and to compare these findings to the ASA classification, an instrument commonly used for quantifying patients' physical status and anesthetic risk.

Background

In the onco-geriatric surgical population it is important to identify patients at increased risk of adverse post-operative outcome to minimize the risk of over- and under-treatment and improve outcome in this population.

Methods

263 patients ≥70 years undergoing elective surgery for solid tumors were prospectively recruited. Primary endpoint was 30-day morbidity. Pre-operatively TUG was administered and ASA-classification was registered. Data were analyzed using multivariable logistic regression analyses to estimate odds ratios (OR) and 95% confidence intervals (95%-CI). Absolute risks and area under the receiver operating characteristic curves (AUC's) were calculated.

Results

164 (62.4%) patients (median age: 76) underwent major surgery. 50 (19.5%) patients experienced major complications. 50.0% of patients with high TUG and 24.8% of patients with ASA≥3 experienced major complications (absolute risks). TUG and ASA were independent predictors of the occurrence of major complications (TUG:OR 3.43; 95%-CI = 1.13–10.36. ASA1 vs. 2:OR 5.67; 95%-CI = 0.86–37.32. ASA1 vs. 3&4:OR 11.75; 95%-CI = 1.62–85.11).

AUC_{TUG} was 0.66 (95%-CI = 0.57–0.75, p<0.001) and AUC_{ASA} was 0.58 (95%-CI = 0.49–0.67, p = 0.09).

Conclusions

Twice as many onco-geriatric patients at risk of post-operative complications, who might benefit from pre-operative interventions, are identified using TUG than when using ASA.

Introduction

With the ageing of our society, the onco-geriatric surgical population is expected to increase. Currently 40% of all malignancies occur in patients over 70 years of age and the majority of patients undergoing surgery for a solid tumor are elderly[1–3]. Roughly 40% of this onco-geriatric surgical population can be considered to be frail[4,5], which is defined as 'a loss of resources in several domains of functioning' resulting in increased vulnerability to stressors[6].

Frail onco-geriatric patients are at an increased risk of adverse outcome due to complications[7]. These patients need to be identified pre-operatively to allow the effective implementation of preventive measures, to minimize the risk of over- and under-treatment and improve outcome in this population. The comprehensive geriatric assessment (CGA) has been introduced to identify frailty in geriatric oncology[8,9]. Unfortunately, CGA is time consuming and hence difficult to utilize in a busy clinical surgical practice. To easily identify which patients are at risk of post-operative complications and might benefit from further assessment and pre-operative interventions[10,11], time saving screening tools need to be investigated.

The American Society of Anesthesiology classification (ASA) is a well-known classification that quantifies the pre-operative physical status and gives an estimation of a patient's anesthetic risk[12]. Studies show opposing results regarding the predictive value of high ASA-scores for post-operative morbidity and mortality[4,13–16]. So far, the ASA-classification has not been proven predictive of post-operative outcome in onco-geriatric patients.

The "Timed Up and Go" (TUG) is a tool that has been made available for the purpose of identifying frail elderly by quantifying functional mobility[17]. It is an easy to administer measure of functional status. The TUG has extensively been studied in community dwelling elderly [18–22] and it was found to predict the risk of early death in onco-geriatric patients receiving chemotherapy[23]. The TUG was also investigated in cohorts of surgical patients. The TUG predicts long-term functional outcome in patients undergoing hip surgery[24,25]. In patients undergoing major cardiovascular or abdominal surgery, the TUG successfully predicted discharge institutionalization and post-operative delirium[26,27]. Data on the predictive value of the TUG in the onco-geriatric surgical population are lacking.

Our aim was to determine the predictive value of the TUG in a prospective cohort study and to compare this to the ASA-classification, a widely used instrument in the field of surgical oncology.

Methods

2.1 Ethics statement

Approval from the National Research Ethics Service Committee North West—Greater Manchester Central and the Medical Ethical Committee from Leiden University Medical Center was obtained and all included patients gave written informed consent. There was no financial incentive to the contributing centers for entering patients into the present study and no funding was acquired. PREOP is registered at the Dutch Trial register (Trial ID: NTR1567).

2.2 Design

A multicenter, prospective cohort study was designed to investigate Pre-operative Risk Estimation for Onco-geriatric Patients (PREOP). The PREOP-study is an international study conducted to analyze several screening tools with regard to short term post-operative outcome. Recruitment took place in 6 different countries at 11 medical centers between September 2008 and January 2012. To reduce the possibility of selection bias and the influence of intercenter variability, medical centers that included less than 10 patients were excluded from analysis. Centers participated actively during different periods of time, depending on the availability of research staff, explaining the relatively small number of included patients considering the long inclusion period.

2.3 Patients

A cohort of cancer patients aged ≥70 who were candidate for elective surgery under general anesthesia, were invited to take part by the local coordinator. Patients requiring emergency surgical management (within 24 hours) were excluded from this study. This international study sample comprised a series of 302 patients. Medical centers that included less than 10 patients were excluded from analysis, which resulted in the analysis of 263 patients (Table 1).

2.4 Endpoints

The primary endpoint was morbidity during the first 30 days after surgery. Morbidity was registered using the Clavien-Dindo classification, a scale ranking severity of complications from 'any deviation from the normal post-operative course without the need for pharmacological treatment or surgical, endoscopic and radiological interventions' (grade one) to 'death of a patient' (grade five)[28]. Morbidity was dichotomized into minor (Clavien-Dindo grade one and two) and major complications (Clavien-Dindo grade three to five). Subsequently, a dichotomous variable was created for morbidity during the first 30 days after surgery: "no/minor" versus "major" complications. Secondary endpoints were 30-day mortality, length of hospital stay, amount of days spent in the Intensive Care Unit (ICU) and the number of additional specialists involved in patient care. The secondary endpoints were dichotomized and cut off points were fixed at >7 days for length of stay after surgery, which was considered prolonged length of stay (LOS), >1 day admission at the ICU and >3 additional specialists involved in patient care.

2.5 Pre- and peri-operative data

Within two weeks prior to the surgical procedure, the TUG was administered as part of a larger test battery. TUG measures the time a person needs to get up out of a chair, walk three meters

Table 1. Number of patients per center included in statistical analysis.

Center	Number of patients
S. Orsola Malphighi Hospital, Bologna, Italy	119 (45.2%)
University Medical Center Groningen, Groningen, The Netherlands	45 (17.1%)
San Martino University Hospital, Genua, Italy	20 (7.6%)
Regional University Hospital of Patras, Patras, Greece	31 (11.8%)
The Highfield Hospital, Manchester, United Kingdom	19 (7.2%)
S. Maria Hospital, Perugia, Italy	15 (5.7%)
Clinical Center Nis, Nis, Serbia	14 (5.3%)
Total	263

doi:10.1371/journal.pone.0086863.t001

and return to the chair[17]. This is measured in seconds with a handheld stopwatch. Patients performed the TUG two times and for each patient, the mean of the two time measurements was calculated. Based on literature and the distribution of the mean values in the current study population, a score of less than or equal to 20 seconds on the TUG was considered a normal score[26]. The ASA-classification, ranging from 'a normal healthy patient' (ASA1) to 'moribund, i.e. not expected to survive 24h with or without surgery' (ASA5), was determined by an anesthesiologist. The patients with score ASA 3 and ASA4 were combined for analysis.

Pre-operative information regarding living situation, pre-operative hemoglobin level, nutritional status and comorbidity was recorded.

Nutritional status was defined according to the following definitions[29]: Normal nutritional status.

Mildly impaired nutritional status: >5% weight loss in 3 months or food intake less than 50–75% of their normal requirements in the past week.

Moderately impaired nutritional status: >5% weight loss in 2 months or BMI 18.5–20.5 + poor overall condition or food intake 25–60% of their normal requirements in the past week.

Severely impaired nutritional status: >5% weight loss in 1 month (>15% in 3 months) or BMI <18.5 + poor overall condition or food intake 0–25% of their normal requirements in the past week.

Peri-operative data contained type of surgery (dichotomized into minor and major surgery), duration of anesthesia and blood loss during surgery.

At every participating center data were collected by a research nurse.

2.6 Statistical analysis

In a univariable logistic regression the odds ratios (OR) and 95% confidence intervals (95%-CIs) were assessed for the presence of a major complication for each of the baseline characteristics including the TUG, ASA-score and TUG and ASA-score combined (TUG+ASA). When combining TUG and ASA-score, we divided this variable into three categories: 1) normal TUG and ASA1 or ASA2; 2) high TUG or ASA≥3; 3) high TUG and ASA≥3. We focused on the results on high TUG and ASA≥3 compared to both normal values. All ORs and 95%-CIs were adjusted for medical center, as there were large differences between the participating centers regarding the number of patients included and the type of performed surgeries. To further adjust for contributing factors, all baseline characteristics were added to the center-adjusted model, including TUG or ASA or TUG+ASA. A variable was selected for multivariable analysis when a significant OR with a minimal change of OR of 10% was observed in comparison with the center-adjusted univariable model containing TUG, ASA or TUG+ASA. The same procedure was repeated for the secondary endpoints. Sensitivity and specificity of the TUG, ASA and TUG+ASA were calculated for the primary outcome measure. To make an accurate estimation of a patient's risk for a certain outcome, absolute risks were calculated [30]. The area under the receiver operating characteristic curves (AUCs) together with 95%-CIs were calculated for the TUG, ASA and TUG+ASA, if applicable. P-values < 0.05 were considered statistically significant. Data analysis was performed using IBM SPSS Statistics 20.0.

Results

3.1 Patient characteristics

The median age of this cohort was 76 years (Range: 70–96) and 66.5% of patients were female (Table 2). The majority of surgical procedures were laparotomies (n = 156; 59.3%) and breast cancer surgeries (n = 76; 28.9%) (Table 2). Types of malignancies treated by means of a laparotomy were colorectal cancer (n = 86), gastric cancer (n = 21), pancreatic cancer (n = 14),

Table 2. Characteristics of 263 patients ≥70 years undergoing surgery for a solid tumor.

Variable	Value[a]
Age (years)[b]	76 (73–81)
Gender	
Female	175 (66.5%)
Male	88 (33.5%)
Living situation	
Independent/family	258 (99.2%)
Residential care/nursing home	2 (0.8%)
Nutritional status	
Normal	171 (67.6%)
Mildly impaired	62 (24.5%)
Moderately & severely impaired	20 (7.9%)
Comorbidities (n)[b]	3 (2–4)
Hemoglobin level	
≥12g/dl	154 (62.9%)
<12g/dl	91 (37.1%)
Surgery	
Minor	99 (37.6%)
Breast cancer treatment (± lymph node)	76 (28.9%)
Excision malignancies of soft tissue, skin and/or lymph node	16 (6.1%)
Thyroidectomy	4 (1.5%)
Remaining	3 (1.1%)
Major	164 (62.4%)
Laparotomy	156 (59.3%)
Laparoscopic approach of G.I. or G.U. tumors	5 (1.9%)
Excision soft tissue sarcoma and vulvectomy	3 (1.1%)
Duration anesthesia (h)[b]	2.6 (1.5–4.0)
Blood loss during surgery (dl)[b]	1.0 (1.0–2.0)
TUG (s)[b]	11.3 (8.2–16.5)
TUG	
≤20.0 seconds	220 (84.0%)
>20.0 seconds	42 (16.0%)
ASA-score	
1	23 (8.8%)
2	121 (46.5%)
3	109 (41.9%)
4	7 (2.7%)
TUG+ASA	
TUG≤20 + ASA<3	129 (49.8%)
TUG>20 + ASA≥3	26 (10.0%)

[a]Valid percentages were calculated when data were not available from all patients.
[b]Values are median and first and third quartiles.

doi:10.1371/journal.pone.0086863.t002

cholangio-, gallbladder- and papilla of Vater carcinoma (n = 8), ovarian cancer (n = 6), liver metastases of colon cancer (n = 6), and other solid tumors (n = 14). One patient underwent a laparotomy for both colon and renal cell carcinoma. The majority of patients (62.4%) underwent major surgery. The median TUG in our sample was 11.3 seconds (Q_1-Q_3 8.2–16.5). A

total of 220 patients (84.0%) completed the TUG within 20 seconds. The majority of patients were classified as ASA2 (n = 121; 46.5%) and ASA3 (n = 109; 41.9%). A total of 129 patients (49.8%) had both a normal TUG and ASA<3 (Table 2).

3.2 Primary outcome measure

Complications occurred in 123 patients (46.8%) and of these patients, 50 patients developed major complications (Table 3). Compared to women (12.7%), men (33.3%) were at higher risk of developing major complications post-operatively (OR 3.50; 95%-CI = 1.67–7.34; p = 0.001) (Table 4), even when corrected for minor or major surgery (OR 2.29; 95%-CI = 1.06–4.97; p = 0.04).

The absolute risk for patients with high TUG to develop major complications was 50%, in contrast for patients with normal TUG which was 13.6% (Tables 4 & 5). Almost all patients that developed major complications and had a normal TUG underwent major surgery (n = 26; 89.7%). After adjustment for nutritional status and minor or major surgery, patients with a high TUG had a 3.43 times higher risk of developing major complications within 30 days post-operatively as compared to patients with normal TUG (95%-CI = 1.13–10.36; p = 0.03) (Table 5). Sensitivity of a high TUG was 42.0% and specificity was 89.8%. The AUC was 0.66 (95%-CI = 0.57–0.75; p<0.001).

A total of 24.8% of patients classified as ASA3 or ASA4 developed major complications (Tables 4 & 5). From the patients classified as ASA1 or 2 who did develop major complications post-operatively, 19 (90.5%) underwent major surgery. Patients classified as ASA2 had a 5.67 times higher risk of experiencing major complications compared to patients labeled as ASA1 (95%-CI = 0.86–37.32; p = 0.07), when adjusted for nutritional status and minor or major surgery. Patients classified as ASA3 or ASA4 had a 11.75 times higher risk of major complications

Table 3. Outcomes.

Outcome measure	Patients (n = 263)[a]
Complications	
No	140 (53.2%)
Any	123 (46.8%)
Major	50 (19.5%)
Mortality	
No	253 (96.6%)
Yes	9 (3.4%)
Readmission	
No	236 (91.8%)
Yes	21 (8.2%)
Length of stay >7 days	
No	128 (49.0%)
Yes	133 (51.0%)
Length of stay on ICU >1 day	
No	216 (82.4%)
Yes	46 (17.6%)
>3 additional specialists involved	
No	211 (82.7%)
Yes	44 (17.3%)

[a]Valid percentages were calculated when data were not available from all patients.

doi:10.1371/journal.pone.0086863.t003

Table 4. Univariable association between patient characteristics and no/minor and major complications.

Variable	Major complication (n = 263)[a]	Univariable OR (95% CI)[b]
TUG		
≤20.0 seconds	29 (13.6%)	1
>20.0 seconds	21 (50.0%)	**4.86 (1.82–13.00)**
ASA-score		**P = 0.001**[c]
1	2 (9.1%)	1
2	19 (16.0%)	**9.77 (1.58–60.61)**
3&4	28 (24.8%)	**25.92 (3.97–169.47)**
TUG+ASA		
TUG≤20 + ASA<3	12 (9.5%)	1
TUG>20 + ASA≥3	12 (46.2%)	**9.06 (2.49–32.96)**
Age (years)[d]	78 (74–82)	1.05 (0.98–1.12)
Gender		
Female	22 (12.7%)	1
Male	28 (33.3%)	**3.50 (1.67–7.34)**
Living situation		
Independent/family	49 (19.4%)	[e]
Residential care/nursing home	0 (0%)	
Nutritional status		**P<0.001**[c]
Normal	18 (10.7%)	1
Mildly impaired	22 (36.7%)	**4.55 (2.03–10.23)**
Moderately & severely impaired	8 (42.1%)	**5.00 (1.51–16.54)**
Comorbidities (n)[d]	4 (3–6)	**1.66 (1.34–2.05)**
Hemoglobin level		
≥12g/dl	24 (16.0%)	1
<12g/dl	21 (23.6%)	1.21 (0.57–2.53)
Surgery		
Minor	4 (4.0%)	1
Major	46 (29.1%)	**7.32 (2.38–22.49)**
Duration anesthesia (h)[d]	3 (2.2–5.0)	**1.27 (1.08–1.50)**
Blood loss during surgery (dl)[d]	2.0 (1.0–3.0)	**1.36 (1.10–1.69)**

[a]Valid percentages were calculated when data were not available from all patients.

[b]Bold = statistically significant.

[c]Overall significance.

[d]Values are median and first and third quartiles.

[e]Due to small numbers of patients living residential care/nursing home, the living situation could not be included in the logistic regression.

doi:10.1371/journal.pone.0086863.t004

compared to patients classified as ASA1 (95%-CI = 1.62–85.11; p = 0.02) (Table 5). Sensitivity of ASA≥3 was 57.1% and specificity was 58.5%. The AUC was 0.58 (95%-CI = 0.49–0.67, p = 0.09).

A total of 46.2% (n = 12) of patients with both a high TUG and ASA≥3 developed major complications, compared to 9.5% (n = 12) of patients with a normal TUG and ASA<3 (p<0.001) (Tables 4 & 5). Patients with both high TUG and ASA≥3 had a 5.34 times higher risk of developing major complications compared to patients with a normal TUG and ASA<3 (95%-CI = 1.23–23.29; p = 0.03), when adjusted for nutritional status and minor or major surgery (Table 5). Sensitivity was 50.0% and specificity was 89.1%. The AUC was 0.70 (95%-CI = 0.57–0.83; p = 0.002).

Table 5. Multivariable association of TUG and ASA with regard to major complications, prolonged LOS and >3 specialists involved in patient care.

	Major complication		Stay >7 days		>3 specialists involved	
	%[a]	OR (95% CI)[b]	%[a]	OR (95% CI)[c]	%[a]	OR (95% CI)[c]
**TUG**		p = 0.03		p = 0.03		p = 0.002
≤20.0s (n = 214)	13.6%	1	47.3%	1	11.7%	1
>20.0s (n = 42)	50.0%	3.43 (1.13–10.36)	70.0%	4.21 (1.14–15.58)	45.0%	5.39 (1.85–15.77)
**ASA**		p = 0.04		Univariable OR NS		p = 0.002
1 (n = 22)	9.1%	1	65.2%		8.7%	1
2 (n = 119)	16.0%	5.67 (0.86–37.32)	43.0%		7.7%	2.45 (0.35–17.46)
3&4 (n = 113)	24.8%	11.75 (1.62–85.11)	55.3%		27.7%	14.23 (1.87–108.25)
**TUG+ASA**		p = 0.03		p = 0.04		p<0.001
TUG≤20 + ASA<3 (n = 126)	9.5%	1	43.4%	1	4.8%	1
TUG>20 + ASA≥3 (n = 26)	46.2%	5.34 (1.23–23.29)	66.7%	5.21 (1.10–24.73)	54.2%	29.56 (6.21–140.68)

[a]Absolute risks; Valid percentages were calculated when data were not available from all patients.
[b]Adjusted for center, minor/major surgery and nutritional status.
[c]Adjusted for center, gender, minor/major surgery and duration of anesthesia.

doi:10.1371/journal.pone.0086863.t005

3.3 Secondary outcome measures

3.3.1 30-day mortality. Nine patients died post-operatively (30-day mortality rate: 3.4%) (Table 3), all these patients developed major complications prior to death. Three patients died of a pulmonary embolism, three patients died of sepsis, two died of advanced neoplastic disease and one passed away after myocardial infarction. In a univariable logistic regression analysis the TUG and ASA were not predictive of 30-day mortality. The combined TUG and ASA variable was predictive of 30-day mortality in a univariable logistic regression analysis (OR 26.6; 95%-CI 1.79–396.59). Due to low numbers per cell, no multivariable logistic regression analysis was performed for mortality.

3.3.2 Length of stay. After surgery, 133 patients (51.0%) stayed over 7 days in hospital (Table 3) and from these patients, 127 (95.5%) underwent major surgery. The absolute risk for patients with a high TUG to have a prolonged LOS was 70% (n = 28), compared to 47.3% (n = 104) for patients with a normal TUG. The contributing factors in the multivariable logistic regression model for the secondary endpoints were gender, minor or major surgery and duration of anesthesia. In this multivariable logistic regression analysis, patients with a high TUG had a 4.21 times higher risk of prolonged LOS (95%-CI = 1.14–15.58; p = 0.03) (Table 5). The AUC was 0.56 (95%-CI = 0.49–0.63; p = 0.10).

A total of 15 patients (65.2%) with ASA1 had a prolonged LOS and 13 of these patients (86.7%) underwent major surgery. A total of 43.0% (n = 52) classified as ASA2 and 55.3% (n = 63) classified as ASA3 or ASA4 had a prolonged LOS. The majority of these patients underwent major surgery (n = 50 (96.2%) and n = 61 (96.8%) respectively). Prolonged LOS could not be predicted by high ASA-classification in the univariable model (ASA1 vs. 2: OR 0.71; 95%-CI = 0.24–2.10; p = 0.54. ASA1 vs. 3&4: OR 1.53; 95%-CI = 0.50–4.71; p = 0.46) so no multivariable analysis was performed.

A total of 56 patients (43.4%) with a normal TUG and ASA<3 had a prolonged LOS, compared to 16 patients (66.7%) with both a high TUG and ASA≥3. The majority of these patients underwent major surgery as well (n = 52 (92.9%) and n = 14 (87.5%) respectively). In the multivariable logistic regression analysis, patients with both a high TUG and ASA≥3 had a 5.21 times higher risk of prolonged LOS (95%-CI = 1.10–24.73) (Table 5). The AUC was 0.58 (95%-CI = 0.51–0.65; p = 0.03).

3.3.3 Length of stay at Intensive Care Unit. A total of 46 patients (17.6%) required more than one day admission at the ICU ($Q_3 = 1$) (Table 3). All of these patients underwent major surgery. In a univariable logistic regression analysis it was found that neither TUG nor ASA, nor the combined TUG and ASA variable were predictive of a longer stay at the ICU (TUG $p = 0.08$; ASA1 vs. 2 $p = 0.40$; ASA1 vs. 3&4 $p = 0.05$; TUG+ASA $p = 0.06$). Therefore, no multivariable logistic regression analyses were performed.

3.3.4 Number of specialists involved. In 44 patients (17.3%), additional care from more than 3 specialists ($Q_3 = 3$) was required (Table 3). Compared to patients with a normal TUG, relatively more patients with a high TUG needed care from more than 3 specialists (n = 25 (11.7%) and n = 18 (45.0%) respectively). The multivariable logistic regression analysis showed a 5.39 times higher chance to need care from more than 3 specialists in case of a high TUG (95%-CI = 1.85–15.77; $p = 0.002$) (Table 5). The AUC was 0.66 (95%-CI = 0.56–0.76; $p = 0.001$).

Only 2 of the patients with ASA1 (8.7%) required care from more than 3 specialists, in patients with ASA2 this number was 9 (7.7%) and in patients classified as ASA3 or 4, this number was 31 (27.7%). Only patients classified as ASA3 or 4 were over 14 times more likely of requiring additional care from more than 3 specialists (ASA1 vs. 2: OR 2.45; 95%-CI = 0.35–17.46; $p = 0.37$. ASA1 vs. 3&4: OR 14.23; 95%-CI = 1.87–108.25; $p = 0.01$) (Table 5). The AUC was 0.68 (95%-CI = 0.59–0.76; $p<0.001$).

In 54.2% (n = 13) of patients with both a high TUG and ASA$\geq$3, care from more than 3 specialists was required. In patients with a normal TUG and ASA<3, this was 4.8% (n = 6). Patients with both a high TUG and ASA$\geq$3 were 29.56 times more likely of requiring additional care from more than 3 specialists (95%-CI = 6.21–140.68; $p<0.001$). The AUC was 0.76 (95%-CI = 0.68–0.84; $p<0.001$).

Discussion

The use of TUG and ASA as screening tools for short-term post-operative outcome in onco-geriatric surgical patients was investigated. Multivariable analysis showed a prognostic ability of TUG, ASA and TUG and ASA as a combined prognostic tool with regard to the occurrence of major complications within 30 days after surgery. Far more patients at risk of post-operative complications, who might benefit from pre-operative interventions, were identified using the TUG than when using ASA: the absolute risk for patients with high TUG to develop major complications was 50%, while the absolute risk for patients with ASA3 or 4 was 24.8%. The specificity of the TUG was high (89.8%), and the AUC_{TUG} was better than the AUC_{ASA}. The TUG and ASA as a combined variable showed no added value.

A considerable number of patients (n = 123; 46.8%) experiencing complications within 30 days after surgery was recorded, of which 50 (40.7%) developed major complications. Other studies investigating onco-geriatric surgical patients have found a high incidence of post-operative morbidity as well[4,14]. The high morbidity rates emphasize the importance of using pre-operative screening tools to predict short-term post-operative outcome. Moreover, these results point out the urgent need for pre-operative optimization of a substantial percentage of onco-geriatric patients.

In a prospective study among patients $\geq$75 years old undergoing major elective abdominal surgery, multivariable analysis of the predictive value of a high TUG (>20.0 seconds) for post-operative delirium showed a hazard ratio of 4.8. A total of 47.6% of patients with a high TUG suffered from post-operative delirium, compared to only 18.5% of patients with a normal TUG [26]. Robinson et al. found a 13 times higher risk of discharge to an institutional care facility, i.e. nursing home or rehabilitation center, for geriatric surgical patients with a high TUG

(≥15.0 seconds)[27]. In onco-geriatric patients undergoing chemotherapy, a TUG over 20 seconds was found to be a risk factor of death within six months[23]. These data show promising results regarding the use of the TUG as a screening tool in several sets of geriatric patients; to our knowledge this is the first study investigating on the predictive value of the TUG in an onco-geriatric surgical population.

The TUG is a well validated measure, which gives a reflection of a person's muscle strength, mobility and coordination. It is reproducible and proved to be predictive of outcome in the setting of the present large international cohort. However, the cut-off point for the TUG varies greatly between studies, making it difficult to compare outcome and stressing the importance of reporting the used cut-off point. The wide range in cut-off points could be depending on the characteristics of the studied population[18]. Factors as age, whether subjects are hospitalized or community-dwelling and off course the type of outcome measure, are all of influence on the appropriate cut-off point of the TUG score for specific cohorts. An established cut-off point cannot be generalized to an entire population, the lack of a uniform cut-off point for the TUG should therefore be accepted.

Data on ASA predicting the post-operative course have often been studied in colorectal surgical patients, with conflicting results. In a set of colorectal cancer surgical patients, patients with ASA≥3 as a measure of comorbidity were at an increased risk of 30-day mortality and experiencing surgical complications[13]. In octogenarians undergoing colorectal cancer surgery, Tan et al. found patients classified as ASA≥3 being at increased risk of post-operative morbidity[15] and Heriot et al. identified high ASA as a risk factor of 30-day mortality. Patients classified as ASA3 had a 2.86 times higher risk of dying within the first 30 days after surgery and in patients classified as ASA4 or ASA5 this risk increased to 6.08[16]. In a similar population of elderly colorectal cancer patients, however, high ASA was not identified as a risk factor of post-operative complications[4]. This is in keeping with a broader population of onco-geriatric surgical patients, where high ASA was not found to be predictive of post-operative morbidity or mortality[14].

The discrepancy between these results could partly be explained by the interrater variability, which is a main disadvantage of the use of ASA as a screening tool[31]. In the onco-geriatric surgical population, where the majority of patients is classified as ASA2 or ASA3 (Table 2)[4], it is difficult to rely on ASA in order to make a distinction between patients at risk of post-operative complications and patients who are not. This suggests that ASA, which is the combination of comorbidity and the clinician's impression of a patient's functional status, might be not a valid measure to be a decisive screening tool in the onco-geriatric surgical population.

The risk of 30-day mortality could not be predicted by TUG nor ASA in the current cohort, which could be explained by lack of power as calculation of the sample size was based on 30-day morbidity. A limitation of the study was that PREOP did not focus on long-term outcome. It is known that post-operative complications increase long-term mortality rates in elderly patients undergoing major surgery[32], suggesting long-term mortality rates as a better outcome measure than short-term mortality[33]. Nevertheless, it endorses post-operative morbidity as an appropriate endpoint for the geriatric population. The association between post-operative morbidity and long-term mortality in the onco-geriatric population remains to be confirmed.

The current results show that the TUG is a more useful screening tool than ASA to identify those patients most at risk of adverse outcome. Providing extra pre-operative care and prehabilitation to patients with a poor TUG performance may improve the performance on the TUG and thus improve post-operative outcome[34]. This is also emphasized by the ability of TUG to predict the extra need of healthcare post-operatively, shown by the prolonged LOS and the increased number of specialists involved in patients with a high TUG. To optimize the process

of screening for elderly at risk of major post-operative complications, more screening tools should be investigated and compared to the results of TUG and ASA. A recent suggestion is that a combination of screening tools, with different areas of attention, could provide a better predictive value regarding the risk of post-operative morbidity[35]. The final results of a comparison between other instruments aimed at predicting the risk of post-operative complications are awaited.

The PREOP-study is a large multicenter study, which is both a strength and a limitation. Some centers included few patients and patient selection as an explanation for these small number of patients is plausible. We intercepted this by excluding centers who included less than 10 patients. The possibly positive selection bias would, however, certainly not make our findings less likely. The great strength of our multicenter study is the broad generalizability of our results to the onco-geriatric surgical population.

The present analysis suggests that the routine use of the TUG as a screening tool in the onco-geriatric surgical population is of clinical relevance as it is capable of selecting the majority of patients at risk of post-operative complications. Efficiency entails providing the extra pre-operative care to those who will benefit most and within this scope, the TUG could be of great importance.

Acknowledgments

The authors want to thank the following contributing centers for acquisition of data:

S. Orsola Malphighi Hospital, Bologna, Italy
University Medical Center Groningen, Groningen, The Netherlands
San Martino University Hospital, Genua, Italy
Regional University Hospital of Patras, Patras, Greece
The Highfield Hospital, Manchester, United Kingdom
S. Maria Hospital, Perugia, Italy
Clinical Center Nis, Nis, Serbia
Metaxa Cancer Hospital, Piraeus, Greece
Leiden University Medical Center, Leiden, The Netherlands
St. Helens Hospital, St. Helens, United Kingdom
Roger Williams Cancer Center, Providence, United States of America

Author Contributions

Conceived and designed the experiments: BLVL RAA. Performed the experiments: MGH BLVL GU IM JS CS NDC EF RAA. Analyzed the data: MGH BLVL GHDB RAA. Wrote the paper: MGH BLVL GU IM JS CS NDC EF GHDB RAA.

References

1. Balducci L, Extermann M. Management of cancer in the older person: a practical approach. Oncologist 2000; 5(3):224–237. PMID: 10884501
2. De werkgroep 'Prevalentie van Kanker' van de Signaleringscommissie Kanker van KWF Kankerbestrijding. Signaleringscommissie Kanker van KWF Kankerbestrijding. Kanker in Nederland: Trends, prognoses en implicaties voor zorgvraag. 2004.
3. Yancik R, Ries LA. Cancer in older persons. Magnitude of the problem—how do we apply what we know? Cancer 1994 Oct 1; 74(7 Suppl):1995–2003.
4. Kristjansson SR, Nesbakken A, Jordhoy MS, Skovlund E, Audisio RA, Johannessen HO, et al. Comprehensive geriatric assessment can predict complications in elderly patients after elective surgery for colorectal cancer: a prospective observational cohort study. Crit Rev Oncol Hematol 2010 Dec; 76 (3):208–217. doi: 10.1016/j.critrevonc.2009.11.002 PMID: 20005123

5. Ronning B, Wyller TB, Seljeflot I, Jordhoy MS, Skovlund E, Nesbakken A, et al. Frailty measures, inflammatory biomarkers and post-operative complications in older surgical patients. Age Ageing 2010 Nov; 39(6):758–761. doi: 10.1093/ageing/afq123 PMID: 20843962
6. Schuurmans H, Steverink N, Lindenberg S, Frieswijk N, Slaets JP. Old or frail: what tells us more? J Gerontol A Biol Sci Med Sci 2004 Sep; 59(9):M962–5. PMID: 15472162
7. Audisio RA, Ramesh H, Longo WE, Zbar AP, Pope D. Preoperative assessment of surgical risk in onco-geriatric patients. Oncologist 2005 Apr; 10(4):262–268. PMID: 15821246
8. Brunello A, Sandri R, Extermann M. Multidimensional geriatric evaluation for older cancer patients as a clinical and research tool. Cancer Treat Rev 2009 Oct; 35(6):487–492. doi: 10.1016/j.ctrv.2009.04.005 PMID: 19481353
9. Extermann M, Hurria A. Comprehensive geriatric assessment for older patients with cancer. J Clin Oncol 2007 May 10; 25(14):1824–1831. PMID: 17488980
10. Rizzo JA, Bogardus ST Jr, Leo-Summers L, Williams CS, Acampora D, Inouye SK. Multicomponent targeted intervention to prevent delirium in hospitalized older patients: what is the economic value? Med Care 2001 Jul; 39(7):740–752. PMID: 11458138
11. Hempenius L, van Leeuwen BL, van Asselt DZ, Hoekstra HJ, Wiggers T, Slaets JP, et al. Structured analyses of interventions to prevent delirium. Int J Geriatr Psychiatry 2011 May; 26(5):441–450. doi: 10.1002/gps.2560 PMID: 20848577
12. Owens WD, Felts JA, Spitznagel EL Jr. ASA physical status classifications: a study of consistency of ratings. Anesthesiology 1978 Oct; 49(4):239–243. PMID: 697077
13. Dekker JW, Gooiker GA, van der Geest LG, Kolfschoten NE, Struikmans H, Putter H, et al. Use of different comorbidity scores for risk-adjustment in the evaluation of quality of colorectal cancer surgery: Does it matter? Eur J Surg Oncol 2012 Jun 14.
14. PACE participants, Audisio RA, Pope D, Ramesh HS, Gennari R, van Leeuwen BL, et al. Shall we operate? Preoperative assessment in elderly cancer patients (PACE) can help. A SIOG surgical task force prospective study. Crit Rev Oncol Hematol 2008 Feb; 65(2):156–163. PMID: 18082416
15. Tan KY, Kawamura Y, Mizokami K, Sasaki J, Tsujinaka S, Maeda T, et al. Colorectal surgery in octogenarian patients—outcomes and predictors of morbidity. Int J Colorectal Dis 2009 Feb; 24(2):185–189. doi: 10.1007/s00384-008-0615-9 PMID: 19050901
16. Heriot AG, Tekkis PP, Smith JJ, Cohen CR, Montgomery A, Audisio RA, et al. Prediction of postoperative mortality in elderly patients with colorectal cancer. Dis Colon Rectum 2006 Jun; 49(6):816–824. PMID: 16741639
17. Podsiadlo D, Richardson S. The timed "Up & Go": a test of basic functional mobility for frail elderly persons. J Am Geriatr Soc 1991 Feb; 39(2):142–148. PMID: 1991946
18. Bischoff HA, Stahelin HB, Monsch AU, Iversen MD, Weyh A, von Dechend M, et al. Identifying a cut-off point for normal mobility: a comparison of the timed 'up and go' test in community-dwelling and institutionalised elderly women. Age Ageing 2003 May; 32(3):315–320. PMID: 12720619
19. Davis DH, Rockwood MR, Mitnitski AB, Rockwood K. Impairments in mobility and balance in relation to frailty. Arch Gerontol Geriatr 2011 Jul-Aug; 53(1):79–83. doi: 10.1016/j.archger.2010.06.013 PMID: 20678816
20. Kim MJ, Yabushita N, Kim MK, Matsuo T, Okuno J, Tanaka K. Alternative items for identifying hierarchical levels of physical disability by using physical performance tests in women aged 75 years and older. Geriatr Gerontol Int 2010 Oct; 10(4):302–310. doi: 10.1111/j.1447-0594.2010.00614.x PMID: 20345433
21. Kim MJ, Yabushita N, Kim MK, Nemoto M, Seino S, Tanaka K. Mobility performance tests for discriminating high risk of frailty in community-dwelling older women. Arch Gerontol Geriatr 2010 Sep-Oct; 51 (2):192–198. doi: 10.1016/j.archger.2009.10.007 PMID: 19939477
22. Rockwood K, Awalt E, Carver D, MacKnight C. Feasibility and measurement properties of the functional reach and the timed up and go tests in the Canadian study of health and aging. J Gerontol A Biol Sci Med Sci 2000 Feb; 55(2):M70–3. PMID: 10737688
23. Soubeyran P, Fonck M, Blanc-Bisson C, Blanc JF, Ceccaldi J, Mertens C, et al. Predictors of early death risk in older patients treated with first-line chemotherapy for cancer. J Clin Oncol 2012 May 20; 30(15):1829–1834. doi: 10.1200/JCO.2011.35.7442 PMID: 22508806
24. Ingemarsson AH, Frandin K, Mellstrom D, Moller M. Walking ability and activity level after hip fracture in the elderly—a follow-up. J Rehabil Med 2003 Mar; 35(2):76–83. PMID: 12691337
25. Laflamme GY, Rouleau DM, Leduc S, Roy L, Beaumont E. The Timed Up and Go test is an early predictor of functional outcome after hemiarthroplasty for femoral neck fracture. J Bone Joint Surg Am 2012 Jul 3; 94(13):1175–1179. doi: 10.2106/JBJS.J.01952 PMID: 22760384

26. Brouquet A, Cudennec T, Benoist S, Moulias S, Beauchet A, Penna C, et al. Impaired mobility, ASA status and administration of tramadol are risk factors for postoperative delirium in patients aged 75 years or more after major abdominal surgery. Ann Surg 2010 Apr; 251(4):759–765. doi: 10.1097/SLA.0b013e3181c1cfc9 PMID: 20224380
27. Robinson TN, Wallace JI, Wu DS, Wiktor A, Pointer LF, Pfister SM, et al. Accumulated frailty characteristics predict postoperative discharge institutionalization in the geriatric patient. J Am Coll Surg 2011 Jul; 213(1):37–42; discussion 42–4. doi: 10.1016/j.jamcollsurg.2011.01.056 PMID: 21435921
28. Clavien PA, Barkun J, de Oliveira ML, Vauthey JN, Dindo D, Schulick RD, et al. The Clavien-Dindo classification of surgical complications: five-year experience. Ann Surg 2009 Aug; 250(2):187–196. doi: 10.1097/SLA.0b013e3181b13ca2 PMID: 19638912
29. Kondrup J, Allison SP, Elia M, Vellas B, Plauth M, Educational and Clinical Practice Committee, European Society of Parenteral and Enteral Nutrition (ESPEN). ESPEN guidelines for nutrition screening 2002. Clin Nutr 2003 Aug; 22(4):415–421. PMID: 12880610
30. Dalton JE, Kattan MW. Recent advances in evaluating the prognostic value of a marker. Scand J Clin Lab Invest Suppl 2010 Jul; 242:59–62. doi: 10.3109/00365513.2010.493389 PMID: 20515279
31. Aronson WL, McAuliffe MS, Miller K. Variability in the American Society of Anesthesiologists Physical Status Classification Scale. AANA J 2003 Aug; 71(4):265–274. PMID: 13677221
32. Rutten HJ, den Dulk M, Lemmens VE, van de Velde CJ, Marijnen CA. Controversies of total mesorectal excision for rectal cancer in elderly patients. Lancet Oncol 2008 May; 9(5):494–501. doi: 10.1016/S1470-2045(08)70129-3 PMID: 18452860
33. Dekker JW, van den Broek CB, Bastiaannet E, van de Geest LG, Tollenaar RA, Liefers GJ. Importance of the first postoperative year in the prognosis of elderly colorectal cancer patients. Ann Surg Oncol 2011 Jun; 18(6):1533–1539. doi: 10.1245/s10434-011-1671-x PMID: 21445672
34. Harari D, Hopper A, Dhesi J, Babic-Illman G, Lockwood L, Martin F. Proactive care of older people undergoing surgery ('POPS'): designing, embedding, evaluating and funding a comprehensive geriatric assessment service for older elective surgical patients. Age Ageing 2007 Mar; 36(2):190–196. PMID: 17259638
35. Hamaker ME, Jonker JM, de Rooij SE, Vos AG, Smorenburg CH, van Munster BC. Frailty screening methods for predicting outcome of a comprehensive geriatric assessment in elderly patients with cancer: a systematic review. Lancet Oncol 2012 Oct; 13(10):e437–44. doi: 10.1016/S1470-2045(12)70259-0 PMID: 23026829
